# Dr. Andrew Buck Parsons

**Published:** 1886-01

**Authors:** 


### Obituary.

Dr. Andrew Buck Parsons was born in Fairfax, Vt., November 10, 1822, and died of cerebral hemorrhage at Atlanta, Ga., the 30th of last November.

He received a liberal education, and graduated from the Berkshire Medical College in 1846. The following year he attended lectures at the College of Physicians and Surgeons, New York. Returning to his native town of Fairfax, he practiced his profession there until 1852, when he located in East Randolph, this State. Here it was that the greater part of his life's work was done. A man of correct habits, strict integrity, a vigorous constitution, almost unlimited energy, combined with more than a usual knowledge of his profession, it was not long before he was actively employed with a large and responsible practice.

In 1873, the doctor removed to Jamestown, where he has since resided. For the past few years, he had been in poor health, and spent his winters south. This year, feeling in poorer health than usual, he left, the earlier part of November, for Atlanta, where he died after an illness of a few days.

Dr. Parsons was dignified, yet courteous, in manner, and although positive in his opinions, he was magnanimous, incapable of the little jealousies which so often mar professional life. Owing to his superior skill, sound judgment, and the high estimation in which he was held by his medical brethren, his counsel was largely sought by them.

When the Medical Department of Niagara University was organized, Dr. Parsons was appointed by the trustees one of the examiners.

A wife and two daughters are left to mourn his loss.

